# Transcriptome and Co-Expression Network Analysis Reveals the Molecular Mechanism of Rice Root Systems in Response to Low-Nitrogen Conditions

**DOI:** 10.3390/ijms24065290

**Published:** 2023-03-09

**Authors:** Weiping Wang, Wei Xin, Ning Chen, Fan Yang, Jia Li, Guize Qu, Xingdong Jiang, Lu Xu, Shijiao Zhao, Hualong Liu, Luomiao Yang, Hongliang Zheng, Detang Zou, Jingguo Wang

**Affiliations:** 1 College of Agriculture, Northeast Agricultural University, Harbin 150030, China; 13039819866@163.com (W.W.); xinweineau@163.com (W.X.); neau_chenning@163.com (N.C.); yf201613@163.com (F.Y.); lijia2422@163.com (J.L.); qu2215123581@163.com (G.Q.); jcdingjun@163.com (X.J.); xulu9523@163.com (L.X.); 18345309792@163.com (S.Z.); liuhualongneau@163.com (H.L.); yaochang616@163.com (L.Y.); zhenghongliang008@163.com (H.Z.); zoudtneau@126.com (D.Z.); 2 Key Laboratory of Germplasm Enhancement and Physiology & Ecology of Food Crop in Cold Region, Ministry of Education, Harbin 150030, China

**Keywords:** low-nitrogen conditions, rice (*Oryza sativa*), RNA-seq, WGCNA

## Abstract

Nitrogen is an important nutrient for plant growth and essential metabolic processes. Roots integrally obtain nutrients from soil and are closely related to the growth and development of plants. In this study, the morphological analysis of rice root tissues collected at different time points under low-nitrogen and normal nitrogen conditions demonstrated that, compared with normal nitrogen treatment, the root growth and nitrogen use efficiency (NUE) of rice under low-nitrogen treatment were significantly improved. To better understand the molecular mechanisms of the rice root system’s response to low-nitrogen conditions, a comprehensive transcriptome analysis of rice seedling roots under low-nitrogen and control conditions was conducted in this study. As a result, 3171 differentially expressed genes (DEGs) were identified. Rice seedling roots enhance NUE and promote root development by regulating the genes related to nitrogen absorption and utilization, carbon metabolism, root growth and development, and phytohormones, thereby adapting to low-nitrogen conditions. A total of 25,377 genes were divided into 14 modules using weighted gene co-expression network analysis (WGCNA). Two modules were significantly associated with nitrogen absorption and utilization. A total of 8 core genes and 43 co-expression candidates related to nitrogen absorption and utilization were obtained in these two modules. Further studies on these genes will contribute to the understanding of low-nitrogen adaptation and nitrogen utilization mechanisms in rice.

## 1. Introduction

Rice is one of the most widely grown food crops in the world. Approximately 50% of the world’s population uses rice as their staple food [1]. Rice yield has continued to increase in recent decades, partly due to the application of large amounts of nitrogen fertilizer [2,3]. However, the average utilization efficiency of nitrogen fertilizer in China is only 33% [4]. High nitrogen input and low-nitrogen use efficiency not only increase crop production costs but also lead to the eutrophication of water bodies [5], soil hardening [6], environmental pollution, and many other problems. This has seriously affected the sustainable development of rice production [7]. Therefore, reducing nitrogen fertilizer application and improving nitrogen use efficiency (NUE) are important goals of sustainable agriculture.

Roots are essential for the growth and development of plants [8]. The morphological and physiological characteristics of root systems closely relate to nitrogen absorption and utilization capabilities. To elaborate, having more and deeper root systems increases the efficiency of absorbing nitrogen from deep soils [9]. Previous studies have shown that nitrogen supply levels have a significant effect on the structure and physiological properties of rice roots [3]. In general, proper nitrogen deficiency promotes root growth and improves nitrogen absorption [9,10], while an excessive nitrogen supply inhibits root growth and reduces nitrogen absorption [11,12]. Although the effect of nitrogen on rice roots is obvious, the understanding of the molecular regulatory mechanism of nitrogen in regulating root development is still limited. Therefore, understanding the molecular mechanism of nitrogen absorption and the utilization of roots under low-nitrogen conditions is essential to improve the NUE and yield of rice.

Root architecture is regulated by intrinsic factors such as root development genes, transcription factors, small RNAs, and phytohormones and is influenced by some extrinsic factors. The cumulative effect of all these factors ultimately determines root formation [13,14,15]. The root structure of rice includes primary, adventitious, and lateral roots, which are regulated by different genes and transcription factors. For example, *OsNRT2.4* promotes nitrogen absorption and lateral root growth [16], and *OsWOX11* and *OsWOX3A* regulate primary and lateral root development [17,18]. Plant hormones such as auxin, ethylene, and jasmonic acid also regulate the root structure of rice [19,20,21]. RNA sequencing (RNA-seq) is a highly sensitive and powerful transcriptomic tool for the identification of differentially expressed genes (DEGs) [22]. It has been applied to identify the genes that are responsive to nitrogen [23,24,25]. Therefore, it is an important tool for exploring the causes of root growth changes in rice under different nitrogen concentrations.

Weighted gene co-expression network analysis (WGCNA) is a method that is based on the expression data specified module of gene chip or RNA-seq. It uses system biology to understand co-expression networks and explores the associations between genes and target traits [26]. In rice, WGCNA was used to analyze gene expression data from various sources to predict the response of core genes to drought [27], low temperature [28], and salt [29], and the module of co-expressed genes under stress. WGCNA was also adopted to identify the central gene regulating nitrogen metabolism and the transcription factors associated with nitrogen utilization in rice [30,31]. Therefore, it is feasible to use WGCNA to identify the genes that are related to nitrogen utilization in rice.

This paper aims to better understand the morphological changes and molecular mechanism of nitrogen absorption and utilization of rice roots under low nitrogen. To achieve this, we investigated the morphological traits, such as root length, lateral root number, average root diameter, dry weight, nitrogen content, and NUE, of aboveground and underground parts and conducted transcriptome analysis at different time points. The transcriptome analyses indicated that the rice roots adapted to low-nitrogen conditions by regulating the genes related to nitrogen absorption and utilization, carbon metabolism, and root growth and development. Using WGCNA and constructing co-expression modules, the core genes and their co-expression candidates related to nitrogen absorption and utilization were obtained. The identified genes and their transcriptome analysis provided more useful information clarifying the molecular mechanisms of plant adaptation to nitrogen starvation.

## 2. Results

### 2.1. Effect of Low Nitrogen on the Root System of Rice Seedlings

**Figure 1:**
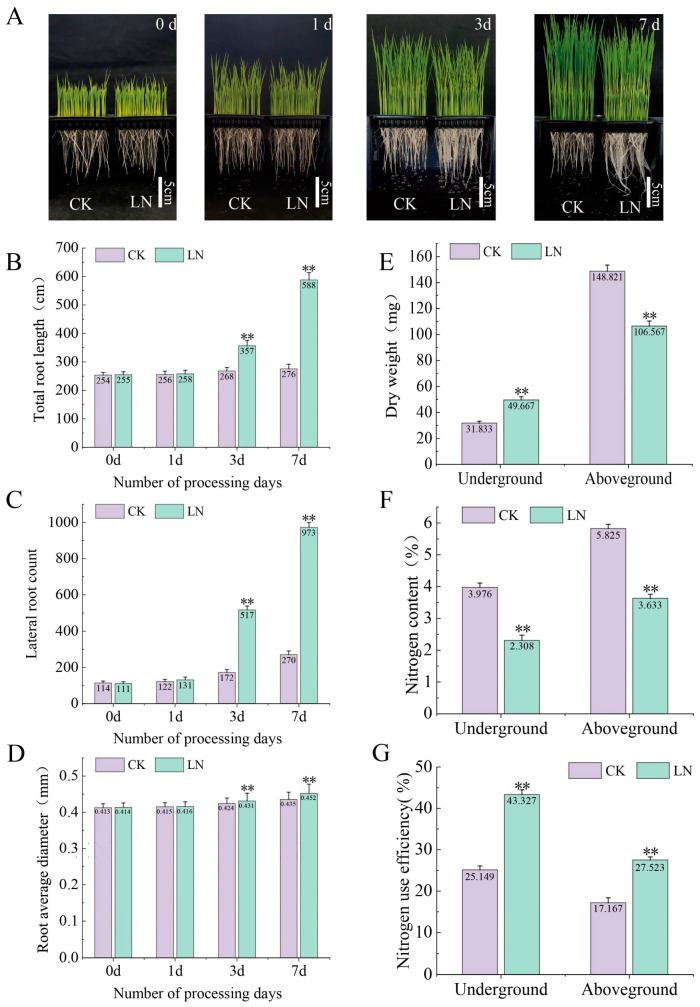
The growth status and morphological and physiological characteristics of rice seedings under low-nitrogen conditions at different time points. (**A**) Seedling growth status. (**B**) Total root length. (**C**) Lateral root count. (**D**) Average root diameter. (**E**) Dry weight of aboveground and underground parts. (**F**) Nitrogen content of aboveground and underground parts. (**G**) Nitrogen use efficiency of aboveground and underground parts. LN and CK represent low-nitrogen treatment and control, respectively. The numbers in the histogram represent the average of each trait. The two asterisks represent significant differences.

Low-nitrogen conditions have a significant effect on the root development of rice. In this study, we measured the total root length, lateral root number, and the average root diameter, dry weight of aboveground and underground parts, and nitrogen content of rice seedlings under low-nitrogen and controlled conditions. It was found that low nitrogen significantly increased the root length, lateral root count, root diameter, and dry weight of underground roots, and significantly decreased the dry weight of aboveground roots and nitrogen content of the whole plant. The NUE was significantly increased under low-nitrogen conditions (Figure 1).

### 2.2. Analysis of Differentially Expressed Genes (DEGs) at Different Time Points

**Figure 2:**
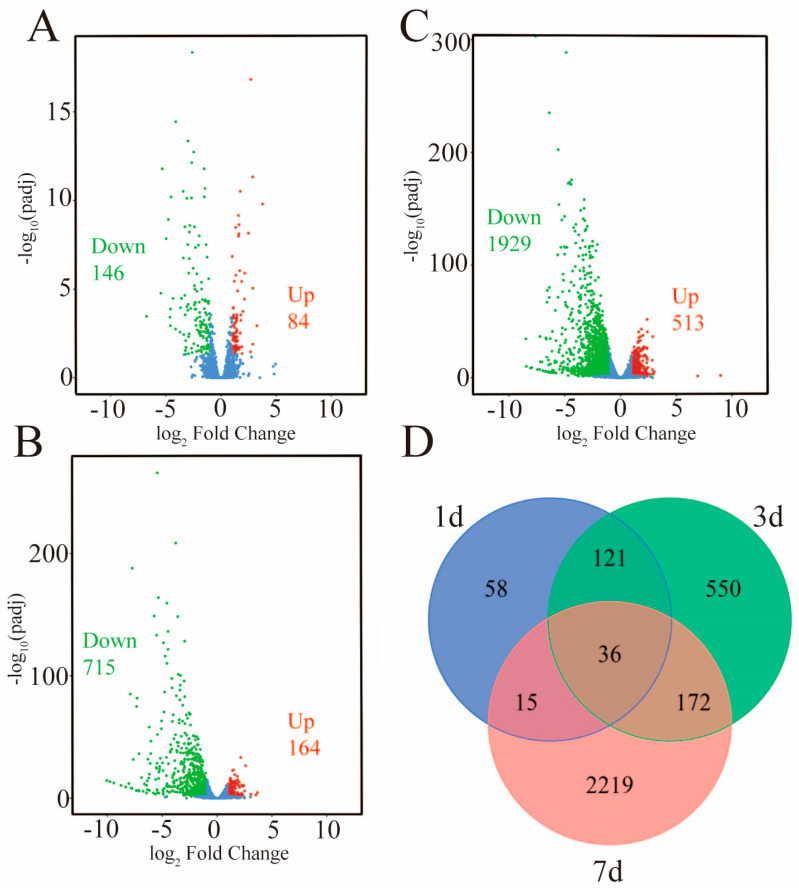
Volcano plot and Venn diagram of DEGs under low−nitrogen conditions. The blue, red, and green dots represent the number of genes that were insignificantly expressed, significantly up−regulated, and significantly down−regulated, respectively. (**A**) Volcano plot of DEGs on the first day under low−nitrogen conditions. (**B**) Volcano plot of DEGs on the third day under low−nitrogen conditions. (**C**) Volcano plot of DEGs on the seventh day under low−nitrogen conditions. (**D**) Venn diagram of DEGs.

RNA-seq generated a total of 183.33 GB of clean readings from 18 root samples at three time points (1 d, 3 d, and 7 d) after low-nitrogen and control treatments. The data from each sample were larger than 7.26 GB with GC contents ranging from 51.4% to 53.1%, and the Q30 base percentage was more than 91.1%. The alignment efficiency of each sample was between 86.1% and 94.1% (Table S1). These results indicate that the quality of the sequencing data was suitable for the subsequent analysis. The DEGs increased with the extension of low-nitrogen treatment time, and a total of 3171 DEGs were identified in this study. Of them, 230, 879, and 2442 DEGs were identified, and the number of up-regulated and down-regulated genes was 84 and 146, 164 and 715, and 513 and 1929 on the first, third, and seventh day after low-nitrogen treatment, respectively (Figure 2A–C). As the nitrogen deficiency progressed, the changes in root structure and biochemistry became increasingly obvious and more stress-responsive genes were induced.

### 2.3. Analysis of GO and KEGG

**Figure 3:**
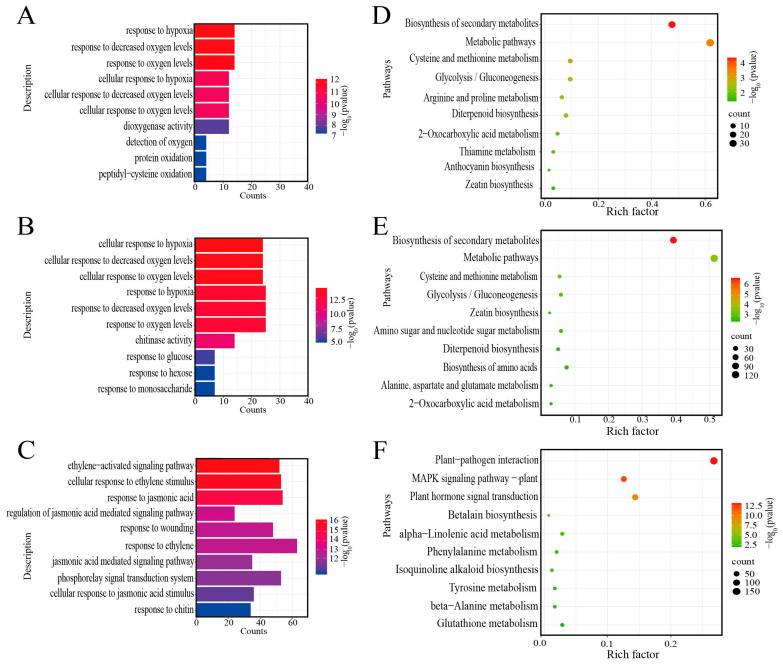
Go and KEGG pathways for the top 10 most enriched DEGs at different time points. (**A**) GO analysis on the 1st day. (**B**) GO analysis on the 3rd day. (**C**) GO analysis on the 7th day. (**D**) KEGG analysis on the 1st day. (**E**) KEGG analysis on the 3rd day. (**F**) KEGG analysis on the 7th day.

The GO terms were similar on the first and third day after low-nitrogen treatment. The response to hypoxia, decreased oxygen levels, and oxygen levels were three of the most significantly enriched GO terms on the first day (Figure 3A). The cellular response to hypoxia, response to decreased oxygen levels, and response to oxygen levels were three of the most significantly enriched GO terms on the third day (Figure 3B). The ethylene-activated signaling pathways, cellular responses to ethylene stimulation, and jasmonic-acid-mediated signaling pathways were three of the most significantly enriched GO terms on the seventh day (Figure 3C). KEGG analyses were conducted to further investigate the specificity of the pathways under low-nitrogen conditions affecting the roots of rice seedings. A total of 1499 DEGs were significantly enriched in 23 pathways (Table S2). Figure 3D–F show the top 10 abundant pathways on the first, third, and seventh day after low-nitrogen treatment, respectively. We also found some significant enrichment of the amino acid metabolic pathways, such as alanine, aspartic acid, glutamate, cysteine, methionine, and phenylalanine metabolism, at different time points (Figure 3D–F). These results indicate that rice may adapt to low nitrogen through phytohormone regulation and amino acid metabolism.

### 2.4. DEGs Related to Nitrogen Absorption and Utilization

**Figure 4:**
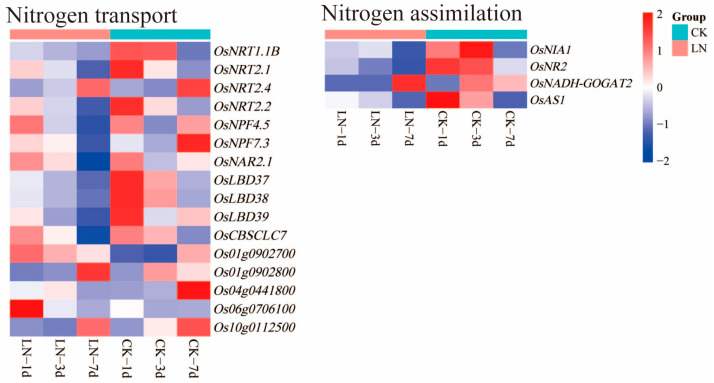
DEGs for nitrogen absorption and utilization under low-nitrogen conditions. LN and CK represent low-nitrogen treatment and control, respectively.

Sixteen DEGs related to nitrogen transport (containing three transcription factors (TFs)) and four DEGs related to nitrogen assimilation were identified in this study. Among the DEGs related to nitrogen transport, *OsLBD37* and *OsLBD38* were down-regulated and two peptide transporters (PTRs) (*Os06g0706100* and *Os01g0902700*) were up-regulated on the first day; *OsNRT2.4* and *Os01g0902700* (PTR) were up-regulated and *OsNRT1.1B*, *OsLBD37*, *OsLBD38*, *Os01g0902800*, and *Os10g0112500* were down-regulated on the third day; and nine DEGs (*OsNRT2.1*, *OsNRT2.2*, *OsNAR2.1*, *OsNPF4.5*, *OsLBD38*, etc.) were down-regulated on the seventh day. Among the DEGs related to nitrogen assimilation, *OsNR2* was down-regulated at all three time points; *OsNIA1*, *OsNADH-GOGAT2*, and *OsAS1* were only down-regulated on the third day (Table S3, Figure 4).

### 2.5. DEGs Related to Carbon Metabolism

**Figure 5:**
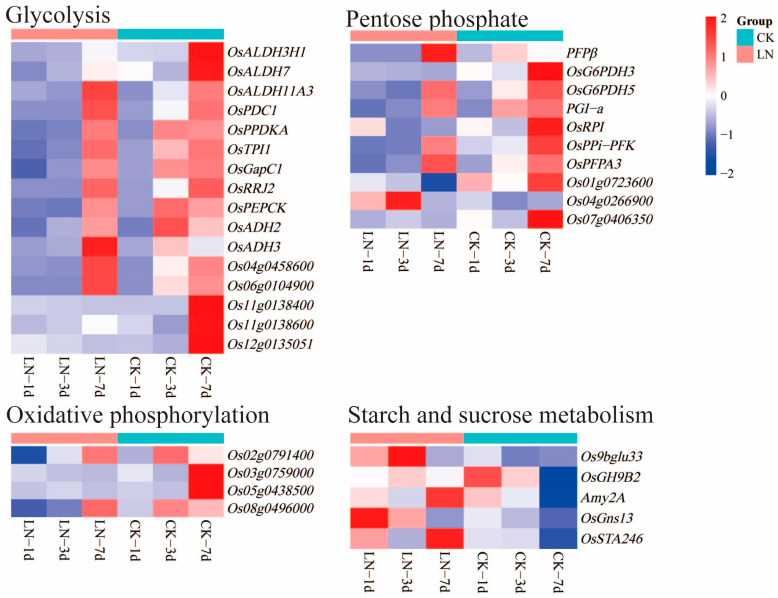
DEGs for carbon metabolism under low-nitrogen conditions. LN and CK represent low-nitrogen treatment and control, respectively.

Sixteen, four, ten, and five DEGs related to glycolysis, oxidative phosphorylation, pentose phosphate, and starch and sucrose metabolism were identified in this study, respectively. Among the DEGs related to glycolysis, *OsPPDKA*, *OsPDC1*, *OsRRJ2*, *OsPEPCK*, and *Os06g0104900* were down-regulated on the first day; eleven DEGs, including *OsALDH11A3*, *OsADH2*, *OsADH3*, *OsPPDKA*, *OsPDC1*, etc., were down-regulated on the third day; *OsADH3* was up-regulated on the seventh day. Among the DEGs related to oxidative phosphorylation, *Os02g0791400* was down-regulated on the first day; *Os08g0496000* was down-regulated on the third day; and *Os03g0759000* and *Os05g0438500* were down-regulated on the seventh day. Among the DEGs related to pentose phosphate, *OsPPi-PFK* was down-regulated on the first day; *OsG6PDH5*, *PGI-a*, *OsPPi-PFK*, and *OsPFPA3* were down-regulated and *Os04g0266900* was up-regulated on the third day; and *OsRPI*, *OsG6PDH3*, *Os01g0723600*, and *Os07g0406350* were down-regulated and *PFPβ* was up-regulated on the seventh day. Among the DEGs related to starch and sucrose metabolism, *OsGns13* was up-regulated on the first and third day; *Os9bglu33* was up-regulated on the third day; and *OsGH9B2*, *Amy2A*, and *OsSTA246* were up-regulated on the seventh day (Table S4, Figure 5).

### 2.6. DEGs Related to Root Growth and Phytohormone

**Figure 6:**
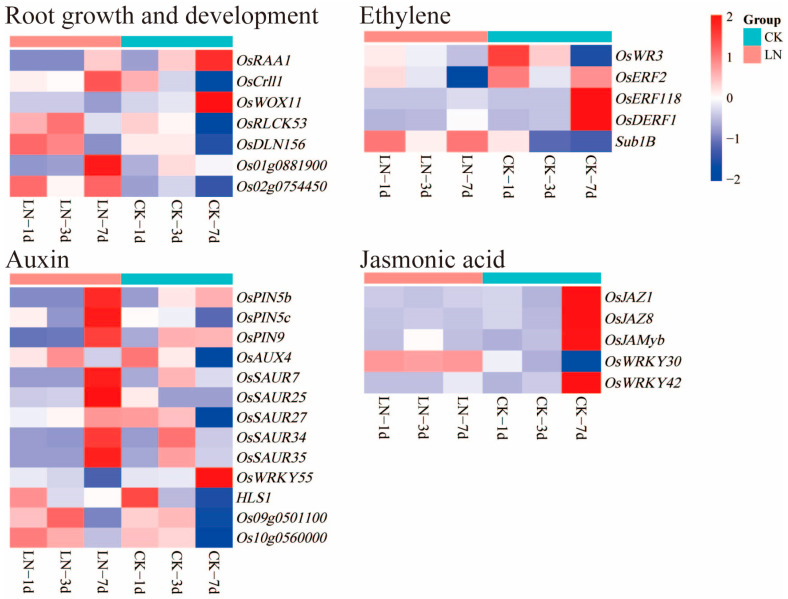
DEGs for root development under low-nitrogen conditions. LN and CK represent low-nitrogen treatment and control, respectively.

Seven root growth and development, thirteen auxins, five ethylenes and five jasmonic acid DEGs related to the regulation of root growth were identified in this study; they contained four, two, five, and five transcription factors, respectively (Table S5). Among the DEGs related to root growth and development, *OsRAA1* and *Os01g0881900* were down-regulated on the third day, while *OsWOX11* was down-regulated and *OsRLCK53*, *OsCrll1*, *OsDLN156*, *Os01g0881900*, and *Os02g0754450* were up-regulated on the seventh day. Among the DEGs related to auxin, *OsPIN5b* was down-regulated on the first day; *OsPIN5b*, *OsPIN9*, *OsSAUR7*, *OsSAUR34*, and *OsSAUR35* were down-regulated on the third day; and *OsWRKY55* was down-regulated and ten DEGs, including *OsSAUR7*, *OsSAUR25*, *OsPIN5c*, *OsAUX4*, *HLS1*, etc., were up-regulated on the seventh day. Among the DEGs related to ethylene, *OsWR3* and *Sub1B* were up-regulated and *OsERF2*, *OsDERF1*, and *OsERF118* were down-regulated on the seventh day. Among the DEGs related to jasmonic acid, *OsJAMyb* was up-regulated on the third day, while *OsWRKY30* was up-regulated and *OsJAZ1*, *OsJAZ8*, *OsJAMyb*, and *OsWRKY42* were down-regulated on the seventh day (Table S5, Figure 6).

### 2.7. Weighted Gene Co-Expression Analysis (WGCNA) of Rice Root Transcriptome under Low-Nitrogen Conditions

After screening the raw data, 25,377 genes were retained to conduct WGCNA, and 14 modules were obtained (Figure S1). The genes related to nitrogen absorption and utilization were found to be mainly distributed in the brown and black modules (Table S6). So, these two modules may be the key modules involved in the low-nitrogen response; they were considered as the candidate functional module for the subsequent co-expression network analysis and key gene mining. The filter condition was in the first 50% of the weight value of the brown module and in the first 30% of the weight value of the black module (Table S7). *OsNRT2.1*, *OsNRT2.2*, *OsNRT2.4*, *OsNPF4.5*, and 4 PTRs (*Os01g0902800*, *Os01g0960900*, *Os03g0235700*, and *Os10g0112500*) were selected as core genes and 43 candidate genes were obtained that may have mechanisms in the regulation of the low-nitrogen response and nitrogen absorption and utilization (Table S8). Among the candidate genes in the brown module, 15 candidate genes, including *Os02g0734400*, *OsSub33*, *DAO*, *OsCYP94D5b*, *OsRLCK163*, etc., were co-expressed with *OsNRT2.4*; *Os06g0177000*, *OsGLP12-2*, *OsGLP12-3*, *OsSultr1;1*, and *prx81* were co-expressed simultaneously with *OsNRT2.1* and *OsNRT2.2*.

**Figure 7:**
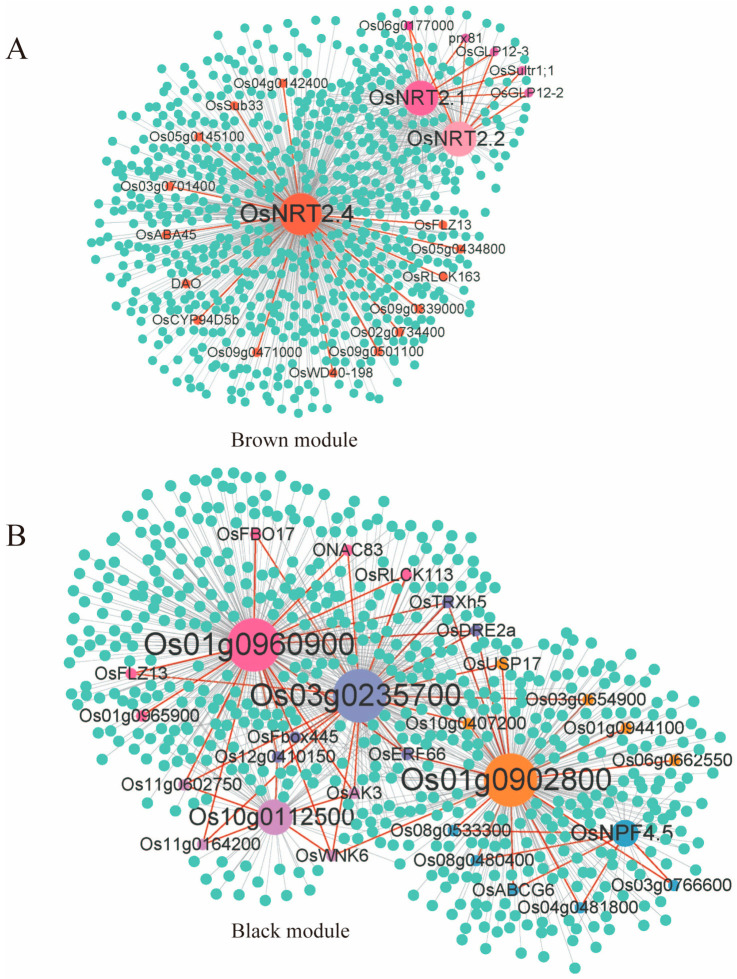
The weighted co-expression network of the genes related to nitrogen absorption and utilization. The large, differently colored circles are the core genes in the figure. The small, differently colored circles are the candidate genes sharing high co-expression intensity with the core genes. (**A**) Brown module. (**B**) Black module.

Among the candidate genes in the black module, *Os03g0766600*, *Os08g0533300*, *Os04g0481800*, *Os08g0480400*, and *OsABCG6* were simultaneously co-expressed with *OsNPF4.5* and *Os01g0902800*; *Os10g0407200*, *OsUSP17*, and *Os03g0654900* were simultaneously co-expressed with two PTRs (*Os01g0902800* and *Os03g0235700*); *Os01g0965900* was co-expressed with *Os01g0960900; Os01g0944100* and *Os06g0662550* were co-expressed with *Os01g0902800*; *OsFBO17*, *OsFLZ13*, *OsRLCK113*, and *ONAC83* were simultaneously co-expressed with two PTRs (*Os03g0235700* and *Os01g0960900*); *Os12g0410150*, *OsAK3*, *Os11g0602750*, and *Os11g0164200* were simultaneously co-expressed with three PTRs (*Os03g0235700*, *Os10g0112500*, and *Os01g0960900*); *OsWNK6* was simultaneously co-expressed with four PTRs (*Os01g0902800*, *Os01g0960900*, *Os03g0235700*, and *Os10g0112500*); *OsFbox445* was simultaneously co-expressed with two PTRs (*Os01g0960900* and *Os03g0235700*); and *OsDRE2a*, *OsTRXh5*, and *OsERF66* were simultaneously co-expressed with three PTRs (*Os01g0902800*, *Os01g0960900*, and *Os03g0235700*) (Figure 7). The expression patterns obtained from the RNA-seq data were verified using quantitative reverse transcriptase polymerase chain reaction(qRT-PCR) for 43 candidate genes under different treatments (low-nitrogen and control nitrogen) for 1 d, 3 d, and 7 d. After normalizing the Ct values using the internal control gene *Actin1*, control nitrogen treatment was used as a reference sample for the low-nitrogen treatment. Based on the log_2_ fold change expression values of the candidate genes, the expression trends of these genes significantly correlated with the results of RNA-seq (Figure S2).

## 3. Discussion

Roots are plants’ main nitrogen-absorbing organ. The adaptive changes in root morphology and physiological characteristics are an important material basis for the effective use of nitrogen by plants [32]. Previous studies have shown that environmental nitrogen content is closely related to rice root morphology, especially under low-nitrogen conditions, in which the root length increases [33,34,35]. In this study, there were no obvious phenotypic changes in rice root under low-nitrogen treatment on the first day; however, the root length, lateral root count, and root diameter of rice significantly increased on the third and seventh day, and the nitrogen use efficiency (NUE) of rice significantly increased, especially underground. Root elongation is an important performance indicator of improved NUE in crops because deeper roots can improve nitrogen absorption from deep soil and reduce nitrogen loss to the environment [9]. Accordingly, these findings suggest that increasing the root length is a strategy allowing plants to cope with low-nitrogen conditions. Compared with the control, the dry weight of rice aboveground and the plant nitrogen content decreased, but the dry weight of rice underground increased under low-nitrogen conditions. This demonstrated that rice may preferentially supply nutrients to the underground parts to promote root growth and maintain life-sustaining activities under nutrient-deprived conditions. Recently, with the development of high-throughput sequencing, transcriptome technology has been widely used to analyze the molecular mechanisms of plant growth and development, nutrient absorption and utilization, and stress tolerance [36,37,38]. However, most previous studies focused on the changes in rice at a single time point under low-nitrogen conditions, ignoring the change process of rice adaptation to low-nitrogen conditions [25,33,39]. Therefore, in this study, we analyzed the transcriptome of rice roots at different time points in the seedling stage under low-nitrogen conditions to explore the molecular mechanism of rice adaptation to nitrogen deficiency.

Inorganic nitrogen is absorbed and transported by specific transfer proteins, such as nitrate transporter (NRT) and ammonium transporter (AMT). Many of the nitrogen transporters of rice, such as *OsNRT1.1B*, *OsNRT2.1*, *OsNRT2.2*, *OsNPF4.5*, *OsNPF7.3*, and *OsNAR2.1* and the three peptide transporters (PTRs) (*Os10g0112500*, *Os01g0902800*, and *Os04g0441800*) were down-regulated in this study. The expressions of two dual-affinity nitrate transporters (*OsNRT1.1B* and *OsNRT2.4*), two high-affinity nitrate transporters (*OsNRT2.1* and *OsNRT2.2*), two low-affinity nitrate transporters (*OsNPF4.5* and *OsNPF7.3*), and one nitrate transporter chaperone protein (*OsNAR2.1*) were regulated by the NO_3_^−^ concentration [40,41,42,43,44]. The expressions of *OsNRT2.1*, *OsNRT2.2*, and *OsNAR2.1* were up-regulated by NO_3_^−^, and the expressions of *OsNRT2.1* and *OsNRT2.2* were significantly down-regulated in the *OsNAR2.1* knockdown mutants [45]. Therefore, it is possible that the down-regulated expression of *OsNAR2.1* led to the down-regulated expression of *OsNRT2.1* and *OsNRT2.2* under low-nitrogen conditions. *OsNRT2.4* and two PTRs (*Os06g0706100* and *Os01g0902700*) were up-regulated in this study. A previous study found that the number and length of lateral roots of rice and the total nitrogen absorption would be reduced by knocking out *OsNRT2.4* [16]. Transcription factor families perform vital roles in responding to the nitrogen source supply [46]. The expressions of *OsLBD37*, *OsLBD38*, and *OsLBD39* of the LOB transcription factors family, which negatively regulate NO_3_^−^ absorption [47], were down-regulated in this study. These results suggest that rice may enhance the active absorption of nitrogen by regulating nitrogen transport proteins and transcription factors and increase the absorption of nitrogen by promoting root growth to adapt to low-nitrogen conditions. Interestingly, some nitrogen assimilation genes of rice, such as *OsNIA1*, *OsNR2*, *OsAS1*, and *OsNADH-GOGAT2*, were down-regulated in this study, which may have been caused by feedback inhibition. Moreover, the accumulation of glutamate inhibits the expression of nitrate reduction and transport genes. Therefore, under low-nitrogen conditions [48,49], rice may adjust nitrogen transport and assimilation by affecting the glutamate cycle to improve NUE.

Nitrogen stress caused changes in gene expression related to the carbon metabolism in rice. The assimilation pathways of carbon and nitrogen are closely related [50]. Carbon metabolism provides adenosine triphosphate (ATP), a reducing agent, and carbon skeleton for nitrogen metabolism. Similarly, nitrogen metabolism provides the organic substances needed for carbon metabolism, such as enzymes and proteins [51,52,53]. Harmonizing the balance between carbon and nitrogen is essential for healthy plant growth, development, and yield formation [54,55]. For many species, nitrogen deficiency leads to increased root growth and redirects the carbon assimilated to underground tissue [56]. The expressions of several enzymes involved in the synthesis and metabolism of sucrose and starch (*OsGH9B2*, *Amy2A*, *OsGns13*, *Os9bglu33*, and *OsSTA246*) were up-regulated, which may be related to root-growth-oriented carbon allocation, because sucrose stimulates the formation of lateral roots [57]. Root starch plays a role in root development and response to nitrate availability [58]. Therefore, rice probably promoted root development by promoting the accumulation of starch and sucrose in the roots under low-nitrogen conditions. In this study, it was observed that the DEGs in glycolysis, oxidative phosphorylation, and pentose phosphate pathways were generally down-regulated. In particular, the expressions of *Os06g0104900* (malate dehydrogenase) and *OsGapC1* (glyceraldehyde-3-phosphate dehydrogenase), which are involved in the glycolytic pathways, were down-regulated. These genes are particularly relevant to the reduction ability. The expressions of two rate-limiting enzymes, *OsG6PDH3* and *OsG6PDH5* (glucose 6-phosphate dehydrogenase), which provide reducing power (NADPH) in the pentose phosphate pathway, were down-regulated. The up-regulated expression of glucose 6-phosphate dehydrogenase probably increases NADPH production [59]. Transcriptome analysis indicated that these genes provide the reduction ability in the glycolytic and pentose phosphate pathways, which may be due to the lower required reduction ability under low-nitrogen conditions. We found, using GO term enrichment analysis, that hypoxic stress occurred in rice roots on the first and third day of the low-nitrogen treatment. This may have been related to the inhibition of oxidative phosphorylation in mitochondria, leading to reduced adenosine triphosphate (ATP) synthesis. A lack of ATP impairs the plasma membrane hydrogen (H^+^) pump ATPase, thus leading to the accumulation of excess reactive oxygen species (ROS) and disrupting normal metabolism [60,61,62]. Therefore, as suggested by this study, rice may promote root growth by regulating the genes related to carbon metabolism to maintain the balance of carbon metabolism and nitrogen metabolism under low-nitrogen conditions.

Low nitrogen induces rice root elongation, and growth is the result of metabolic changes caused by a series of gene expression changes. Among them, the change in auxin plays an important role in the growth and development of rice roots [19]. OsCrll1, the target gene of the auxin response factor, and *OsAUX4*, an auxin input vector, positively regulate the growth and development of rice roots [63,64]. The expressions of OsCrll1 and *OsAUX4* were up-regulated in this study. The asymmetric distribution of auxin efflux transport protein PIN (PIN-FORMED) determines the polar transport of auxin [65]. *OsPIN5b* negatively regulates root growth [66]. In this study, *OsPIN5b* and *OsPIN9* were down-regulated and *OsPIN5c* was up-regulated. One of the pathways for the root itself to synthesize indole-3-acetic acid (IAA) involves acetaldehyde dehydrogenase catalyzing indole-3-acetaldehyde to produce indoleacetic acid. The expressions of acetaldehyde dehydrogenase genes in rice roots, such as *OsALDH3H1, OsALDH7*, and *OsALDH11A3*, were down-regulated under low-nitrogen conditions in this study. The polar transport of IAA moves from the top of the plant to the base, which promotes the initiation and development of lateral roots. According to the changes in the genes related to IAA synthesis and transport, it was speculated that the IAA synthesis ability and the output of rice roots decreased under low-nitrogen conditions, while the IAA input to the roots was enhanced, thus promoting root development. The ethylene-activated signaling pathways, cellular responses to ethylene stimulation, and jasmonic-acid-mediated signaling pathways were three of the most significantly enriched GO terms on the seventh day. The growth of rice adventitious roots was directly controlled by ethylene (ETH), which promotes cell division and elongation growth [20]. The expressions of *OsDERF1* and *OsERF2*, which negatively regulate the ethylene pathway and root growth [67,68], were down-regulated, and the expressions of *OsWR3* and *Sub1B* were up-regulated. Jasmonic acid (JA) promotes the root growth of rice and *Arabidopsis* [21,69]. In this study, we detected many differentially expressed genes involved in jasmonic acid accumulation and signal transduction, such as the up-regulated expression of *OsWRKY30*, the down-regulated expressions of *OsWRKY42*, *OsJAZ8*, and *OsJAZ13*, and the up-regulated expression of *OsJAMyb* followed by its down-regulated expression. A previous study indicated that *OsWRKY30* promotes JA accumulation [70], *OsWRKY42*, *OsJAZ8*, and *OsJAZ13* inhibit JA signaling transduction [71,72,73], and OsJAMyb forms part of the JA signaling pathway [74]. In conclusion, rice roots could improve nutrient absorption efficiency and promote root development by regulating the genes involving plant hormones.

WGCNA is a very useful method for studying the correlation between genes, identifying modules with high phenotype correlation, and mining hub genes in different modules. Some transcription factors and hub genes that responded to nitrate deficiency in rice roots, such as PtaRAP2.11 and *OsNAC36*, were identified using WGCNA [31,75,76]. Therefore, it is feasible to use WGCNA to mine the genes related to nitrogen utilization. We constructed a co-expression network using nitrogen absorption and utilization genes as the core genes. A total of eight core genes were selected in two modules, including *OsNRT2.1*, *OsNRT2.2*, *OsNRT2.4*, *OsNPF4.5*, and four peptide transporters (PTRs) (*Os01g0902800*, *Os01g0960900*, *Os03g0235700*, and *Os10g0112500*). Among them, *OsNRT2.1*, *OsNRT2.2*, *OsNRT2.4*, and *OsNPF4.5* were verified as being related to nitrogen absorption and utilization [41,45,77]. NRT1/PTR (NRT1 PTR family (NPF)) and NRT2 are two major plant nitrate transporter proteins [78]. Some PTR family genes, such as *OsNPF2.2*, *OsPTR6*, and *OsPTR9*, have also been shown to play important roles in nitrogen absorption and utilization [44,79,80]. Therefore, four PTRs (*Os01g0902800*, *Os01g0960900*, *Os03g0235700*, and *Os10g0112500*) could act as core genes and play a role in nitrogen absorption and utilization. In this study, 43 candidate genes co-expressed with core genes were obtained using WGCNA. WGCNA is widely used in transcriptome analyses, such as those performed in research involving growth and development regulation, environmental stress response, yield and quality formation, etc., especially for complex data models [81,82,83]. Some studies have mined and identified many functional genes by constructing a co-expression regulatory network [31,84,85]. Additionally, among the 43 candidate genes, *DAO*, which encodes a growth-hormone-oxidizing dioxygenase, was able to rapidly respond to nitrogen stress [86]; *OsSultr1;1,* the sulfate transporter protein, was regulated by nitrogen in a previous study [87]; *Os08g0533300* was presumed to be ACR5 (accumulation and replication of chloroplasts 5), which has been significantly and positively correlated with plant growth [88]; and *OsWNK6* may be functionally similar to *OsWNK9*, which negatively regulated root elongation [89,90]. These results suggest that these 43 candidate genes may participate in the regulation of the nitrogen uptake and utilization in rice and improve the adaptability of rice to low-nitrogen conditions. In further studies, the function and molecular mechanism of these genes in nitrogen uptake and utilization will be analyzed.

## 4. Materials and Methods

### 4.1. Plant Material

Long Japonica 31, a variety of Japonica rice, was used in this experiment. The main stem has 11 leaves, the plant height is about 92 cm, the spike length is about 15.7 cm, the number of grains per spike is about 86, and the thousand grain weight is about 26.3 g. We identified Long Japonica 31 as a high-NUE rice variety using pre-screening. The plump seeds were kept in an incubator at 48 °C for 48 h to break the dormancy. The seeds were then sown on a 96-well PCR plate, with one seed per well. The seeds were sterilized by soaking them in a 1% sodium hypochlorite solution for 30 min. The sterilized seeds were washed 3 times using distilled water and then placed in an incubator at 30 °C for 2 days to germinate. The germinated seeds were incubated in a greenhouse (22.0 °C/20.0 °C, 10 h in the day/14 h at night). Ammonium nitrate was chosen as the nitrogen source. The concentrations of the low-nitrogen and control conditions were 8 ppm and 40 ppm, respectively. For 5 days, 192 seedlings were incubated under control nitrogen conditions (40 ppm). Then, 96 seedlings were kept in the same solution for 7 days and the remaining 96 seedlings were transplanted to low-nitrogen conditions (8 ppm) for 7 days. The formulation of the hydroponic nutrient solution was based on the conventional nutrient solution formulation of the International Rice Research Institute [53].

### 4.2. Phenotypic Data

After 0 d, 1 d, 3 d, and 7 d of treatment, the total root length, lateral root number, and root diameter were measured using an LA-S plant root analysis system (Microtek Scan Maker 800). The above- and underground parts of the seedlings were collected and dried in an oven at 105 °C for 30 min. The weight of the dry matter was measured. An elemental analyzer (Primacs SNC100-IC-E) was adopted to analyze the nitrogen content of the above- and underground parts, and each sample included 3 replicates, with 10 plants per replicate. The nitrogen use efficiency was calculated using the following formulas:

Nitrogen Accumulation = Dry weight × Nitrogen content

Nitrogen use efficiency (%) = Dry weight/Nitrogen Accumulation × 100

Nitrogen Accumulation = Dry weight × Nitrogen content

Phenotypic data were analyzed descriptively using SPSS 26.0 and Origin 2021b.

### 4.3. RNA Extraction, Illumina Sequencing, and Data Analysis

Eighteen root samples that underwent low-nitrogen treatment and control treatment were collected on the first, third, and seventh days of treatment. This was repeated three times for each sampling point. The total RNA was extracted using the TransZol Up RNA Kit (Beijing, China). The complementary DNA was synthesized from total RNA using the HiFiScrip Gene Synthesis Kit (CWBio, Beijing, China). An Illumina library was constructed according to the manufacturer’s instructions (Illumina, San Diego, CA, USA). High-throughput RNA sequencing was performed using the Illumina HiSeq 2500 platform. The indexing was carried out using HISat v2.1.0 and clean reads were mapped to the genome sequence. The gene matching and FPKM were calculated using FeatureCounts v1.6 software [91]. *p* < 0.05 and |log2FC| > 1 were adopted as the thresholds with which to identify the differentially expressed genes between any two comparative groups using edgeR v3.24.3 [92].

### 4.4. Validation of Candidate Genes

The primer design was performed using Primer Premier 5.0. The total RNA was extracted using the TransZol Up RNA Kit (Beijing, China) and stored at −80 °C. The first-strand cDNA (10 µL) was synthesized according to the instructions for the PrimeScript™ RT Master Mix (Takara Biomedical Technology (Beijing) Co., Ltd., Beijing, China). The BlazeTaq^TM^ SYBR Green qPCR Master Mix 2.0 (GeneCopoeia, GuangZhou, China) and reactions were run on Roche Lightcycler 96 real-time PCR equipment in accordance with the manufacturer’s instructions (Roche Medical Instruments, Basel, Switzerland). Each sample had three technical replicates and *Actian1* was used as an internal control. Finally, the expressions of 43 candidate genes were calculated using the 2^−∆∆CT^ method. All the primers are listed in Table S9.

### 4.5. Functional Enrichment Analysis

The Gene Ontology (GO) and Kyoto Encyclopedia of Genes and Genomes (KEGG) enrichment analyses were performed using the top GO and ClusterProfiler R packages, respectively (default parameters) [93].

### 4.6. Construction and Analysis of Co-Expression Modules and Candidate Genes

In order to construct the gene co-expression network, the WGCNA package in R was chosen to screen the genes [26], and the WGCNA R library was adopted to perform the Dynamic Tree Cut algorithm and dynamic merging. A module was defined as a branch cut of the tree and each module was marked with a unique color. One of them had a confused gene expression of the module, which was not considered for the subsequent analysis. The identification of DEGs related to nitrogen absorption and nitrogen utilization was based on gene annotation. The modules containing many known absorption- and utilization-related genes were designated as the nitrogen-absorption- and nitrogen-utilization-related modules. The genes that co-expressed with genes related to nitrogen absorption and nitrogen utilization in the relevant modules were screened out to construct the co-expression network. The filter condition was in the top 50% of the module weight values. The visualization of the co-expression network was completed using Cytoscape [94] version 3.9.1. In the network, the DEGs with the top 5 weight values co-expressed with each gene related to nitrogen absorption and nitrogen utilization were identified as candidate genes.

## 5. Conclusions

In this study, the morphological analysis of rice root tissues collected at different time points of low- and normal-nitrogen-level treatments showed that root growth and NUE were significantly increased under low-nitrogen treatment. The transcriptomic analysis indicated that nitrogen assimilation in rice is inhibited by feedback regulation and that the pathways involved in carbon metabolism that provide reducing power may also be inhibited due to the lower requirement for reducing power under low-nitrogen conditions. To maintain growth and developmental homeostasis, rice may promote nitrogen absorption by promoting the expression of amphipathic and nitrogen transport proteins and inhibiting the expression of the transcription factors negatively regulating nitrogen transport. It also regulates plant-hormone-related genes, promotes the polar transport of auxin to the root, and inhibits the genes that negatively regulate ethylene and jasmonic-acid-related pathways to promote root growth and development. This work not only provides insight into the molecular mechanism of rice root systems’ response to low-nitrogen conditions, but may also be significant for uncovering the as-of-now unknown nitrogen absorption and utilization genes, thus benefiting the breeding of high-NUE rice cultivars.

## Data Availability

Not applicable.

## Supplementary Materials

The following supporting information can be downloaded at: https://www.mdpi.com/article/10.3390/ijms24065290/s1.

## Abbreviations

NUE: nitrogen use efficiency; GO: Gene Ontology; KEGG: Kyoto Encyclopedia of Genes and Genomes; TFs: transcription factors; DEG: differentially expressed gene; NRT: nitrate transporter; AMT: ammonium transporter; PTRs: peptide transporters; WGCNA: weighted gene co-expression network analysis; RNA-seq: RNA sequencing; qRT-PCR: quantitative reverse transcriptase polymerase chain reaction; IAA: indoleacetic acid; ETH: ethylene; JA: jasmonic acid; ACR5: accumulation and replication of chloroplasts 5.
