# Supplementary material for: Transcriptome and Co-Expression Network Analysis Reveals the Molecular Mechanism of Rice Root Systems in Response to Low-Nitrogen Conditions
Source: Int J Mol Sci. 2023 Mar 9;24(6):5290. doi: 10.3390/ijms24065290 (PMC10048922; doi:10.3390/ijms24065290)
Supplement: Supplementary file 1 [file ijms-24-05290-s001.zip › Captions.pdf]

Supplementary Figure S1. Weighted correlation network analysis of rice roots response to low nitrogen.(A): Dendrogram of gene cluster. (B) : Unsupervised hierarchical clustering dendrograms and heat maps. Colorcoded modules are branches of the clustering tree.

Supplementary Figure S2. Correlation between RNA-seq and qRT-PCR results of candidate genes.

Supplementary Table S1. Summary of read data, cleaning data, percentage of Q30, percentage of GC number, and mappingrate for different samples.

Supplementary Table S2. The top 23 enriched KEGG pathways for DEGs in 1^{st}d、3^{rd}d and 7^{th}d.

Supplementary Table S3. The summary of DEGs related to nitrogen transport and utilization.

Supplementary Table S4. The summary of DEGs related to Carbon metabolism.

Supplementary Table S5. The summary of DEGs related to root growth and phytohormone.

Supplementary Table S6. The distribution of DEGs related to nitrogen absorption and utilization in the module.

Supplementary Table S7. The relationships of Module Network Node.

Supplementary Table S8. The candidate genes in the co-expression network.

Supplementary Table S9. Fluorescent quantitative primers.
