# Supplementary material for: Transcriptome and Co-Expression Network Analysis Reveals the Molecular Mechanism of Rice Root Systems in Response to Low-Nitrogen Conditions
Source: Int J Mol Sci. 2023 Mar 9;24(6):5290. doi: 10.3390/ijms24065290 (PMC10048922; doi:10.3390/ijms24065290)
Supplement: Supplementary file 1 [file ijms-24-05290-s001.zip › Supplementary Figure S1.pdf]

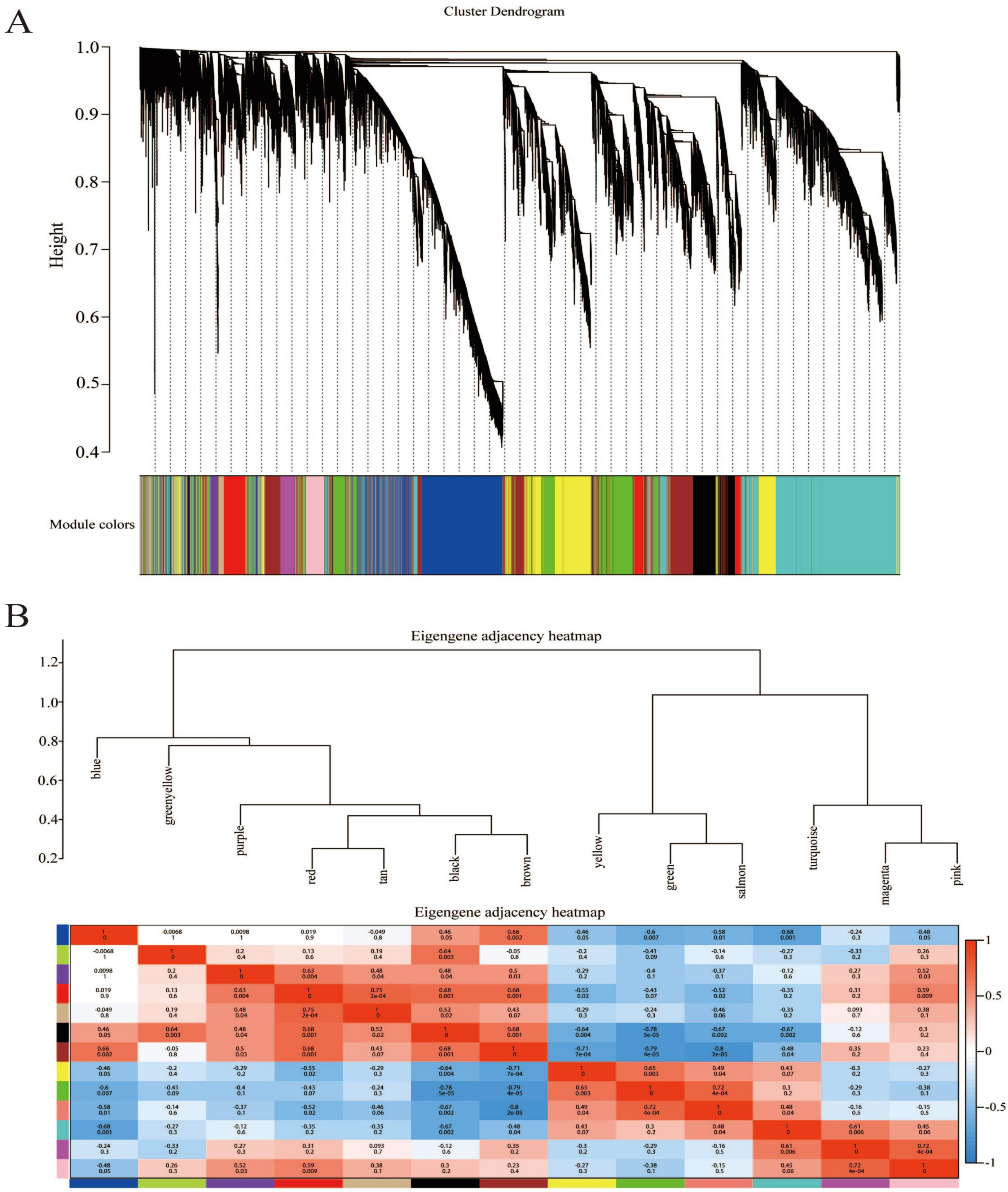

Figure S1. Weighted correlation network analysis of rice roots response to low nitrogen. (A):Dendrogram of gene cluster. (B): Unsupervised hierarchical clustering dendrograms and heat maps. Colorcoded modules are branches of the clustering tree.
