# Supplementary material for: Transcriptome and Co-Expression Network Analysis Reveals the Molecular Mechanism of Rice Root Systems in Response to Low-Nitrogen Conditions
Source: Int J Mol Sci. 2023 Mar 9;24(6):5290. doi: 10.3390/ijms24065290 (PMC10048922; doi:10.3390/ijms24065290)
Supplement: Supplementary file 1 [file ijms-24-05290-s001.zip › Supplementary Table S9.pdf]

Supplementary Table S9 Fluorescent quantitative primers

Gene ID	Upstream primers (5’-3’)	Downstream primers (5’-3’)
Actin	ACCATTGGTGCTGAGCGTTT	CGCAGCTTCCATTCCTATGAA
Os01g0944100	CGATCTCCTCCTTCGTAGTTC	CGAGTAGAAGAGCTTATCTCCG
Os01g0965900	GACAAGAAGCCTCTCAAGAACA	TTATACGGGTAGCGAGATTGTC
Os02g0734400	CGTTTTCCATTACGAATTTGCG	GAGGTAACAACTCCAAATCCGA
Os03g0195800	TCTTCTACAAGTTACACGACCC	GAAGTAGATAGCTGAGTCCACC
Os03g0282100	CTACTGGATATGGCTGCACTAC	CTGTTCAGTTCCAACCCCAT
Os03g0305400	CTTTAGTTCGCTTCGCATGTAA	GGTTAGCGCGAATAGATCAAAG
Os03g0341000	GGTGGAGTACAGCGTTAATGA	CCTTAATCTCCACGGTAAGACA
Os03g0426300	TTGATCCGTCAATGGTGAGAC	CTTGTTCAGCCATGAGGAGTC
Os03g0654900	TCTGCCTGTCAGAGCTGA	ACGACCTAAGAAATCACGGAAT
Os03g0701400	GACACGTCGAGCCTACATTAG	CTGAAATTGTTTGGCTGAGCTC
Os03g0766600	GAATCTGAACCTGGTGATTTCG	TTCACTCGAATCGTCTCATGAT
Os04g0120300	TAATGACAAGTTTGTGGCACAG	CGTTGCCTTGGTGATGTTATAG
Os04g0142400	GCAAATGGGAGACAAGAAACTT	TTTGTATCGATCCATTTTCGCC
Os04g0475600	GTACAGGGAGTTCAAGTACGAC	GGCCTAATGGACATTCTAGTCA
Os04g0476000	TGTGGTATTAAACTTTGTGCGG	GCATTCACAAGGGAAAGTGATT
Os04g0481800	ACTCCTAGTTTGATTACGACGG	TCTGTAGCTCCAAATCTAACGA
Os04g0585900	CAACAATAAAACTGGAAGCCGT	CTCCTTGGGCACTTAGATTTTG
Os04g0619000	GTGATGGCAGATGCGTATATTG	TCTTTCTTCAGGTTATGCGACT
Os04g0632100	CATGTGTTCAAGATGACCCAAC	TAGTTCCATAGACCCTAGCTGT
Os04g0674400	TGATGATAGAAGGGAACATCGG	TAAGTTGCAGTTACCCTAGCAA
Os05g0145100	CGAATAATTCAGTTCGTGCCAA	TCATGCAACCTTGTACAATTGG
Os05g0434800	ACAAAGATCACTGTGACCGATA	GTGACTAACCATGACATGACAG
Os06g0177000	GACATCACCACCAAGATCCACC ACA	TCCTGCTCCTTGTAGTACATTGC GT
Os06g0522300	GGAGTTCACGGAGAGTTACTAC	GTAGCGTTCAGAAGGATGGAG
Os06g0662550	CCACCTACTCTCCTAGAACCTA	GCTAGTATTAGCTGGTGGTAGG
Os07g0190800	TCAGCTCACTGTAACAAGATGG	GAAGTCTATCACCACCAGCTTT
Os08g0428100	CTGGTGTTTGATTGTACAAGGG	TCTGGACCATCACTTAAAAGCT
Os08g0480400	TTCGAGTCAAGTCGTACGATTG	GATGTCGTTGAAGTAGGTGGA
Os08g0533300	TGACCATAAAAAGGGCCTACAT	TCAATTCTGTCAATGACTTGGC
Os09g0339000	GTACAGGTAGCACGTTCTCAG	TATCAACCAACAAATGATCCGG
Os09g0471000	AAGGAAGAGTGGTTTAGCCC	GATGGGAATGTCGGAGAAGTAG
Os09g0501100	AGGAGAACCTGGACGCCAA	ATCATACGAACGCCAAGAAACC
Os10g0407200	AGTGTTCGATTTAATGCGAGTG	CGAAAACACCTACGAATCCTTC
Os11g0160300	GACACTAACAGAAGGACGGTTA	CCTTTTTGTGCTTTGTACGGTA
Os11g0164200	CGGAATCAACAGCATAAGTGAG	GTGAAGCTCGTGGTAGATCTG
Os11g0602750	TTGTTTAGGTTTTCACGCCTTC	AAACAATGCCATCGATACCATG
Os12g0134900	TGCCGCTGAATGGTACTATAAT	AATCATTTTTCTGCCGCTGTAG
Os12g0154800	GATCAATGTGATGAAACTCGCA	GAAGAGCTTGTTTTCTCCGTTT
Os12g0154900	AATGTGATGAAACTTGCAGGTC	GAAGAGCTTGTTTTCTCCGTTT
Os12g0236400	CTCCACTTGGGATTAAGGCTAA	ACCTTTCTGGCATGAAGTTTTC

Os12g0410150	GAAGGGTATATCCAGTGAGGTG	AGCTGAATTTCATGGTTGTGTC
Os12g0478200	TGTGGCTGTGTTTCATTACTTG	ATACATGCCAACAACTAGGAGG
Os12g0594000	AGGGCAACATGTACATGAGTAG	AACAAATCGAAAAATACGCCGC
