# Supplementary figures and images for: Transcriptome and Co-Expression Network Analysis Reveals the Molecular Mechanism of Rice Root Systems in Response to Low-Nitrogen Conditions
Source: Int J Mol Sci. 2023 Mar 9;24(6):5290. doi: 10.3390/ijms24065290 (PMC10048922; doi:10.3390/ijms24065290)

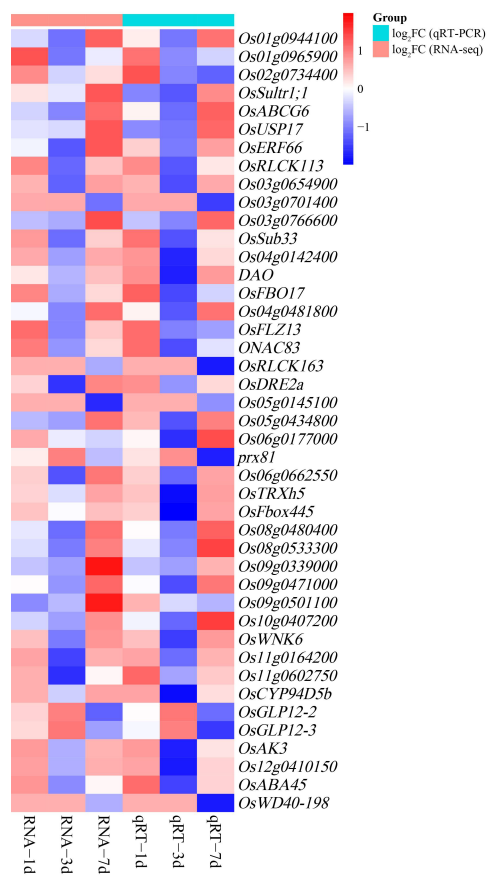

Figure S2. Correlation between RNA-seq and qRT-PCR results of candidate genes.

Supplement: Supplementary file 1 [file ijms-24-05290-s001.zip › Supplementary Figure S2.pdf]
